# The Concept of a Bidirectional Loop of Behavior in Surgery: How Personality and Environment May Reinforce Each Other

**DOI:** 10.1097/AS9.0000000000000702

**Published:** 2026-08-17

**Authors:** Lotte Bouwman, Lieke J. Kerstjens, Vincent Q. Sier, Michael El Boghdady, Maarten Lijkwan, Abbey Schepers, Joris J. Blok

**Affiliations:** From the * Future Surgeon Initiative, Groene Hart Ziekenhuis, Gouda, the Netherlands; † Department of Surgery, Leiden University Medical Center, Leiden, the Netherlands; ‡ Department of General Surgery, Imperial College, London, UK; § Department of Surgery, Albert Schweitzer Ziekenhuis, Dordrecht, the Netherlands; ∥ Department of Surgery, Groene Hart Ziekenhuis, Gouda, the Netherlands

**Keywords:** durability, generations, generational differences, intergenerational relations, personality, surgery, work engagement, work-life balance

## INTRODUCTION

The importance of a safe surgical work environment remains insufficiently recognized in contemporary practice, despite its key role in supporting work performance, job satisfaction, and overall team functioning. Such environments foster positive team culture, effective collaboration, and psychologically safe learning climates.^1–3^ Various forms of mistreatment, including microaggressions, bullying, discrimination, verbal abuse, physical violence, or sexual harassment, continue to occur and contribute to unsafe and toxic workplace cultures.^4^ Recent data indicate that mistreatment is reported by up to 48% of residents and 29% of clinical faculty.^5^ The high workload, clinical responsibility, and frequent exposure to acute and stressful situations inherent to surgery further underscore the necessity of a safe work environment in which teams can function optimally. Working in a toxic environment may not only compromise patient care but also impose a significant psychological burden on healthcare professionals, leading to burnout, psychological distress, and depression.^5–8^

Disruptive behavior, defined as “personal conduct, whether verbal or physical, that negatively affects or has the potential to negatively affect patient care,”^6^ also occurs in surgical environments. It may be perpetuated through systemic normalization, entrenched group culture, informal communication patterns, poorly defined roles, and individual personality traits.^9^

Disruptive behavior has traditionally been explained from 2 perspectives. Some have focused on individual personality traits, whereas others have emphasized the influence of workplace culture and organizational factors. In reality, these explanations are unlikely to be mutually exclusive. Rather than acting independently, personality and environment may continuously influence one another over time, reinforcing either constructive or disruptive patterns of behavior.

The most widely used framework for understanding personality is the Five-Factor Model (Big Five), which categorizes personality traits across 5 core dimensions: extraversion, agreeableness, conscientiousness, negative emotionality (neuroticism), and open-mindedness, resulting in unique personality profiles.^10,11^ Within the Big Five, surgeons tend to display higher average levels of emotional stability, extraversion, and conscientiousness relative to other medical specialists.^12^ Importantly, variation exists across all 5 domains, and these traits should be interpreted within the context of cultural norms, workplace environments, and emotional demands. Additionally, a previous study reported the presence of so-called dark triad traits, including narcissism, in a subset of surgeons, raising the question of whether certain personality characteristics may partially explain disruptive behavior.^13^

Here, we offer a perspective on the interaction between personality traits and workplace culture, exploring whether disruptive behaviors are rooted in individual disposition or shaped and amplified by toxic clinical environments. Most importantly, we introduce the concept of bidirectionality, whereby personality and the surgical environment dynamically interact to reinforce either disruptive or constructive patterns of behavior.

## PERSONALITY VERSUS ENVIRONMENT

Individuals possess unique profiles across the Big Five personality domains, which may influence professional behavior and interpersonal interactions.^14^ A Dutch study demonstrated that surgically interested students, surgical residents, and surgeons scored higher on extraversion, conscientiousness, agreeableness, and open-mindedness, and lower on negative emotions, suggesting a relationship between personality traits and professional environments.^11^ These findings were further supported by a study that reported higher levels of these personality traits across medical students, surgical residents, and surgeons compared with the normative population.^15^

Insights from team selection in extreme environments, such as long-duration space missions, highlight the critical role of individual personality traits in shaping team cohesion, resilience, and task performance.^16^ Using the Big Five taxonomy, astronaut crews with complementary profiles of high conscientiousness, high agreeableness, and low neuroticism have been shown to maintain superior teamwork and adaptive functioning under prolonged stress.^17^ Although a direct parallel cannot be drawn and the comparison should be interpreted with caution, surgical teams may at times operate in high-stakes, high-pressure conditions where cognitive load, rapid decision-making, and stress are common. The space mission example illustrates how personality traits such as conscientiousness, agreeableness, and openness within team members may influence communication, collaboration, and overall team performance.^18^

Beyond individual personality traits, systematic differences also emerge between generations.^19^ Generational differences further underscore the influence of personality traits on the work environment as well as the influence of the work environment on individuals. Recent large-scale research has demonstrated that key Big Five domains vary systematically across surgical generations, with higher conscientiousness and lower negative emotionality scores observed in more experienced surgeons compared with earlier career stages, and distinct patterns of open-mindedness scores among residents relative to students and surgeons.^15^ It remains unclear whether these generational patterns reflect role-based progression, age, or a true generational effect. Current students may come to resemble today’s surgeons as they progress through training, or may retain distinct traits shaped by their original generational context. This is a question that only longitudinal research can answer. However, these findings suggest that surgical work environments and educational structures may both shape and be shaped by evolving personality profiles across career stages, highlighting the importance of generational context.

Moreover, gender differences also influence team composition and dynamics. A cross-sectional study examined sex differences in Big Five personality domains among Swedish general surgeons, revealing that female surgeons scored significantly higher than their male counterparts on several core dimensions of personality.^18^ Female surgeons demonstrated higher levels of conscientiousness, extraversion, agreeableness, and neuroticism compared with male surgeons. These findings suggest that typical gender differences in Big Five measures may also be present to some degree within a highly selected professional cohort, though not necessarily in the same pattern as in the general population.^20^ When considered alongside the traits associated with effective performance in high-pressure teams, the inclusion of women may enhance communication, teamwork, and decision-making in surgical settings. Supporting this perspective, team dynamics in the operating room may also be influenced by gender composition rather than simply the proportion of men or women,^21^ underscoring the importance of diversity in shaping teams.

While previous insights indicate how individual personality traits shape team dynamics, it is equally important to recognize that team culture can, in turn, influence how personality is expressed and adapted. Team culture can modulate the expression and adaptation of personality within high-pressure environments, as illustrated in sports. For example, under coach Alyson Annan, the Dutch national women’s field hockey team operated with a highly competitive culture in which openness, individuality, and psychological safety were often constrained by pressure to succeed. As a result, leading players modified their behavior, communication, and self-expression to align with team norms.^22^ This illustrates how cultural expectations can directly influence how personality traits are expressed in practice. In team sports, leadership style and group norms may modulate the expression of traits like agreeableness, conscientiousness, and emotional stability, with inclusive, trust-based climates promoting adaptive behaviors.^23,24^ This example illustrates that high-stakes work cultures can shape how personality traits manifest in teamwork, communication, and performance, which may also be applicable to the surgical field.

## THE BIDIRECTIONAL LOOP OF BEHAVIOR

**FIGURE 1.**
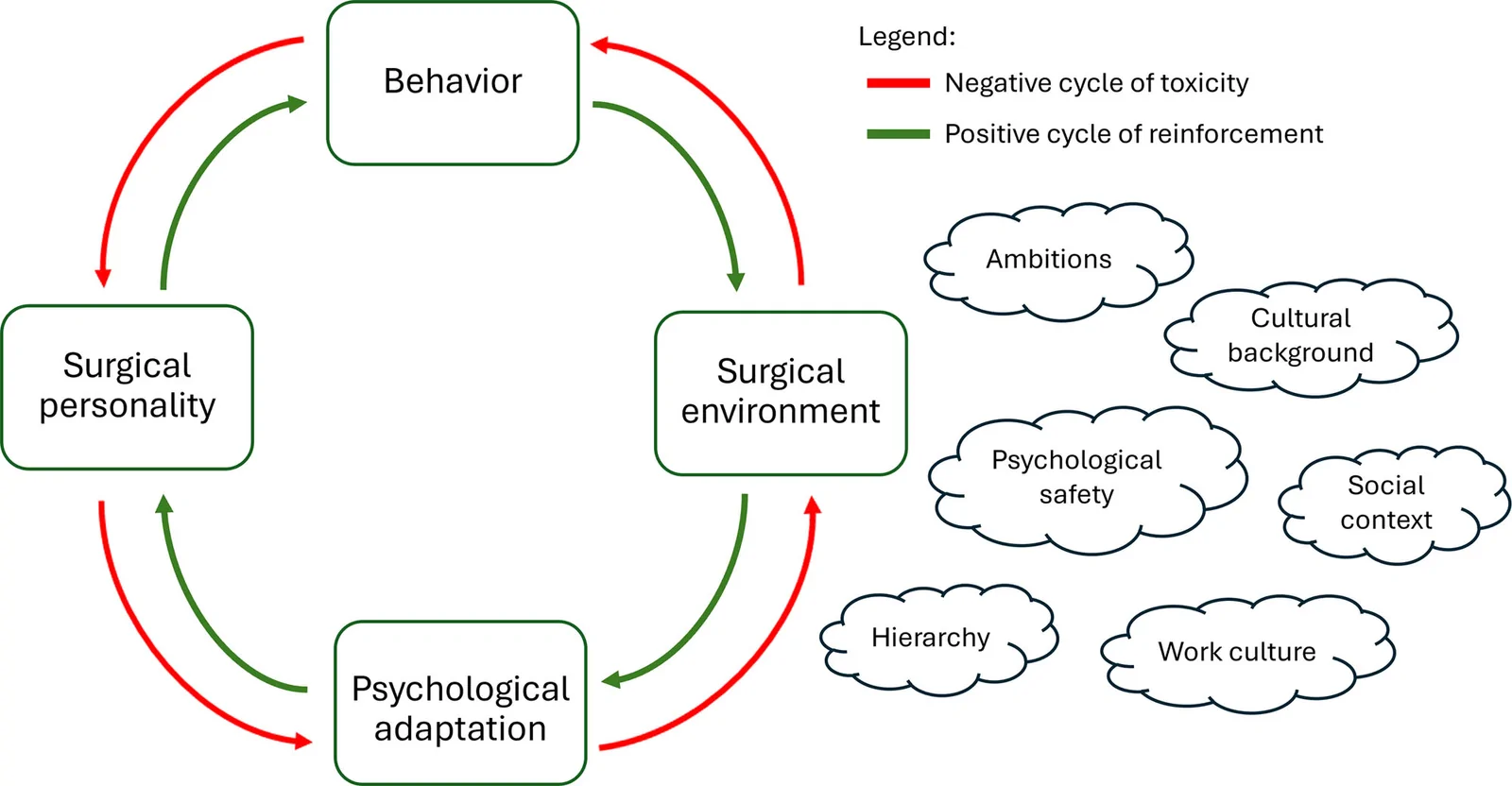
The bidirectional loop of behavior (BLB) concept. Red arrows/lines: negative cycle of toxicity. Green arrows/lines: positive cycle of reinforcement. Various potential factors of influence are demonstrated in the clouds.

Considering both the influence of individual personality traits on team dynamics and the impact of workplace culture on personality expression, the relationship between behavior and the work environment appear inherently bidirectional. Rather than representing a simple linear chain of cause and effect, we introduce the concept that this relationship resembles a loop in which neither personality nor environment acts as the sole instigator (Fig. 1), the bidirectional loop of behavior (BLB). These factors may interact over time in ways that reinforce both environmental culture and behavioral patterns.^25^

However, this interaction is influenced by broader contextual and individual factors, including cultural background and social environment, and individual disposition, and should not be viewed as exclusively cyclical.^25^ Beyond team-level dynamics, workplace behavior may also be shaped by top-down influences, including institutional governance and training systems, which can constrain personality expression and team culture without necessarily being shaped by them in return.^26^ Additionally, not all behavioral change follows gradual cyclical patterns, as structural interventions or acute stressors may rapidly alter behavior through threshold effects, and as such, being linear, asymmetric, and externally driven pathways alongside the previously discussed cyclical dynamics.

The BLB should therefore be seen as a useful heuristic, rather than a universal explanatory model.

If a toxic environment can exacerbate maladaptive personality expression, supportive environments and adaptive personality traits may similarly reinforce one another. Broad personality domains, including extraversion, conscientiousness, agreeableness, open-mindedness, and neuroticism, play a crucial role in determining how individuals perform in the workplace.^25^ For instance, extraversion can enhance communication and facilitate teamwork; however, it may also suppress input from less outspoken members or result in unbalanced interaction patterns. This highlights that no single personality configuration is universally optimal, as team effectiveness depends on contextual demands and team composition. Some traits may benefit from similarity among members (eg, conscientiousness), whereas others may be advantageous when more diverse (eg, open-mindedness), although such effects should be interpreted on a case-by-case basis.

A supportive workplace environment and culture are associated with the development and reinforcement of adaptive personality tendencies, further illustrating the reciprocal relationship between individual characteristics and environmental context. When functioning constructively, this BLB may theoretically help explain the appeal of certain workplaces: it benefits not only individual team members but also strengthens team culture as a whole. This perspective suggests that the BLB may contribute to either constructive or toxic workplace cultures, highlighting its relevance to improving the surgical climate. Although this perspective focuses primarily on team dynamics, the BLB may ultimately influence patient care. Disruptive behaviors are associated with poorer communication, impaired teamwork, and reduced patient safety, whereas constructive workplace cultures may enhance collaboration and ultimately improve (surgical) outcomes.^6^

## CONCLUSIONS

Surgery is a demanding profession, associated with substantial emotional, cognitive, and physical pressures. The interaction between the surgical environment and personality, here described as the BLB, is complex and influenced by multiple factors, including personality traits, personal background, team dynamics, generational differences, and gender distribution. While the concept of the BLB provides a useful heuristic for understanding these dynamics, behavior may also be driven by unidirectional or externally imposed factors that fall outside this cyclical framework. Maladaptive personality expressions may contribute to dysfunctional work environments; however, adverse workplace cultures can likewise amplify such behaviors. This ‘negative’ BLB could be transformed into a ‘constructive’ BLB through mutual reinforcement of supportive leadership, a healthy workplace culture and psychological safety. The real challenge is not resolving a chicken-and-egg dilemma, but recognizing that personality and environment can either perpetuate toxicity or reinforce positive, adaptive surgical cultures.

## ACKNOWLEDGEMENTS

The authors acknowledge the suggestions made by Roderick F. Schmitz. The members of Future Surgeon Initiative are Lotte Bouwman, Lieke J. Kerstjens, Vincent Q. Sier, Abbey Schepers and Joris J. Blok.
